# Partial pressure of end-tidal carbon dioxide successful predicts cardiopulmonary resuscitation in the field: a prospective observational study

**DOI:** 10.1186/cc7009

**Published:** 2008-09-11

**Authors:** Miran Kolar, Miljenko Križmarić, Petra Klemen2, Štefek Grmec

**Affiliations:** 1 Medikmiko-General Practice, Gregorčičeva, 3000 Celje, Slovenia; 2 Faculty of Health Sciences, University of Maribor, Žitna ulica, 2000 Maribor, Slovenia; 3 Centre for Emergency Medicine Maribor, Ulica talcev, 2000 Maribor, Slovenia; 4 University of Maribor, Medical Faculty, Slomškov trg, 2000 Maribor, Slovenia

## Abstract

**Introduction:**

Prognosis in patients suffering out-of-hospital cardiac arrest is poor. Higher survival rates have been observed only in patients with ventricular fibrillation who were fortunate enough to have basic and advanced life support initiated soon after cardiac arrest. An ability to predict cardiac arrest outcomes would be useful for resuscitation. Changes in expired end-tidal carbon dioxide levels during cardiopulmonary resuscitation (CPR) may be a useful, noninvasive predictor of successful resuscitation and survival from cardiac arrest, and could help in determining when to cease CPR efforts.

**Methods:**

This is a prospective, observational study of 737 cases of out-of-hospital cardiac arrest. The patients were intubated and measurements of end-tidal carbon dioxide taken. Data according to the Utstein criteria, demographic information, medical data, and partial pressure of end-tidal carbon dioxide (PetCO_2_) values were collected for each patient in cardiac arrest by the emergency physician. We hypothesized that an end-tidal carbon dioxide level of 1.9 kPa (14.3 mmHg) or more after 20 minutes of standard advanced cardiac life support would predict restoration of spontaneous circulation (ROSC).

**Results:**

PetCO_2 _after 20 minutes of advanced life support averaged 0.92 ± 0.29 kPa (6.9 ± 2.2 mmHg) in patients who did not have ROSC and 4.36 ± 1.11 kPa (32.8 ± 9.1 mmHg) in those who did (*P *< 0.001). End-tidal carbon dioxide values of 1.9 kPa (14.3 mmHg) or less discriminated between the 402 patients with ROSC and 335 patients without. When a 20-minute end-tidal carbon dioxide value of 1.9 kPa (14.3 mmHg) or less was used as a screening test to predict ROSC, the sensitivity, specificity, positive predictive value, and negative predictive value were all 100%.

**Conclusions:**

End-tidal carbon dioxide levels of more than 1.9 kPa (14.3 mmHg) after 20 minutes may be used to predict ROSC with accuracy. End-tidal carbon dioxide levels should be monitored during CPR and considered a useful prognostic value for determining the outcome of resuscitative efforts and when to cease CPR in the field.

## Introduction

Despite all of the progress that has been made in reanimating patients in cardiac arrest over the past half century, resuscitation attempts often fail to restore spontaneous circulation. Consistent and discouraging low survival rates mandate a reassessment of current resuscitative strategies and techniques [1-5]. Overall survival after out-of-hospital cardiac arrest is frequently under 3% [6-8], and so the most common of all decisions after initiation of cardiopulmonary resuscitation (CPR) remains the decision of when to stop. An library of research and guidelines for terminating resuscitative efforts has been developed during the past two decades, and various clinical indicators have been used to determine when CPR efforts should be terminated [8-12]. Capnography (capnometry) potentially represents a useful clinical indicator of death that could guide decisions to terminate resuscitative efforts [8,13]. We sought to evaluate the hypothesis that partial pressure of end-tidal carbon dioxide (PetCO_2_) can predict nonsurvival in an independent cohort of patients suffering out-of-hospital cardiac arrest.

## Materials and methods

A total of 737 patients who suffered a sudden cardiac arrest in the field and were treated by a mobile emergency team were included in the present prospective study. The data were obtained fin the field in Maribor (approximately 200,000 inhabitants). The study was approved by the Ethics Board of the Ministry of Health of the Republic of Slovenia (59/05/00), which granted a waiver of the need for informed consent. Whenever possible, patients who regained consciousness or their relatives were informed of the study after enrollment.

Consistent with the European Union recommendations, we have a single emergency number: 112. In the Centre for Emergency Medicine Maribor there are two prehospital emergency teams and two basic life support teams equipped with defibrillators. In addition, from April till October during the daytime, in Maribor there is a motorcycle rescuer with defibrillation capability; he and the prehospital emergency team are simultaneously dispatched and they rendezvous in the field.

The prehospital emergency team is an advanced life support unit including three members with an adequately equipped road vehicle. The team includes an emergency physician and two registered nurses or medical technicians.

The basic life support team includes two medical technicians or nurses and driver (paramedic). The motorcycle rescuer is a registered nurse or nurse. The prehospital emergency team is routinely dispatched to the field in emergency situations (in case of presumed cardiac arrest, heart attacks, respiratory distress, cerebrovascular incident, trauma, delivery, poisoning and so on). Basic life support and advanced life support are provided using a regional protocol that incorporates European Resuscitation Council standards and guidelines, and clinical algorithms for cardiac resuscitation. After resuscitation, the patient is transferred to the intensive care unit (ICU) of the University Clinical Center, Maribor. Data in accordance with the Utstein criteria, demographic information, medical data and PetCO_2 _values were collected for each patient in cardiac arrest by the emergency physician. Hospital records were used for outcome analysis, which also included assessment of cerebral performance category (CPC) by the intensive care unit specialist. A CPC score of 1 reflects good cerebral performance, CPC scores of 2 and 3 indicate moderate and severe cerebral disability, a CPC score of 4 indicates a comatose, vegetative stage, and CPC score 5 indicates brain death.

All nontraumatic out-of-hospital cardiac arrests in adults older than 18 years in the years from January 1998 to December 2006 were included in the study. Exclusion criteria were documented terminal illness and severe hypothermia (<30°C). We defined return of spontaneous circulation (ROSC) in accordance with the Utstein style ('any ROSC' – palpabile pulse on carotid artery, regardless of duration, and ROSC with admission to hospital). In our analysis and comparison, we consider only those patients with ROSC on admission to hospital (defined as having a stable blood pressure when the prehospital resuscitation team was dismissed by the ICU team).

An endotracheal tube was immediately connected to the capnometer. We measured PetCO_2 _continuously and recorded it during resuscitation, beginning with intial postintubation PetCO_2 _(first PetCO_2 _value obtained) and ending with the final PetCO_2 _value at admission to the hospital or termination of resuscitation attempts. Measurements of PetCO_2 _were taken using the sidestream method with the infrared capnometer integrated into the LIFEPACK 12 defibrillator monitor (Physio Control, Medtronic Inc., Redmond, WA, USA) or with BCI Capnocheck Model 20600A1 (BCi International, Waukesha, WI, USA).

Continuous data are expressed as median (range) and other data are expressed as mean ± standard deviation. Proportions were reported with 95% confidence interval. Analysis for caterogical variables were performed using χ^2 ^test (with Yates correction, if appropriate) and exact Fisher test. Comparisons between groups were performed using *t*-test (normal distribution) and Mann-Whitney test (normality test failed). Sensitivity, specificity, and positive predictive value (PPV) and negative predictive value (NPV) were calaculated using standard formulae. For each value, receiver operating characteristic (ROC) curves were obtained. The ROC curve depicts the relation between true positive results (number of predicted deaths among those who actually died) and false positive results (number of predicted deaths among those who actually survived) for each score. The greater the area under the ROC curve (AUROC), the better the predictive value of PetCO_2_.

Analyses of independent predictors for ROSC and survival from univariate analysis were performed using multivariate logistic regression.

The null hypothesis was considered to be rejected at *P *values less than 0.05 in all tests. For statistical analysis we used SPSS12.01 software (SPSS Inc., Chicago, IL, USA).

## Results

**Figure 1:**
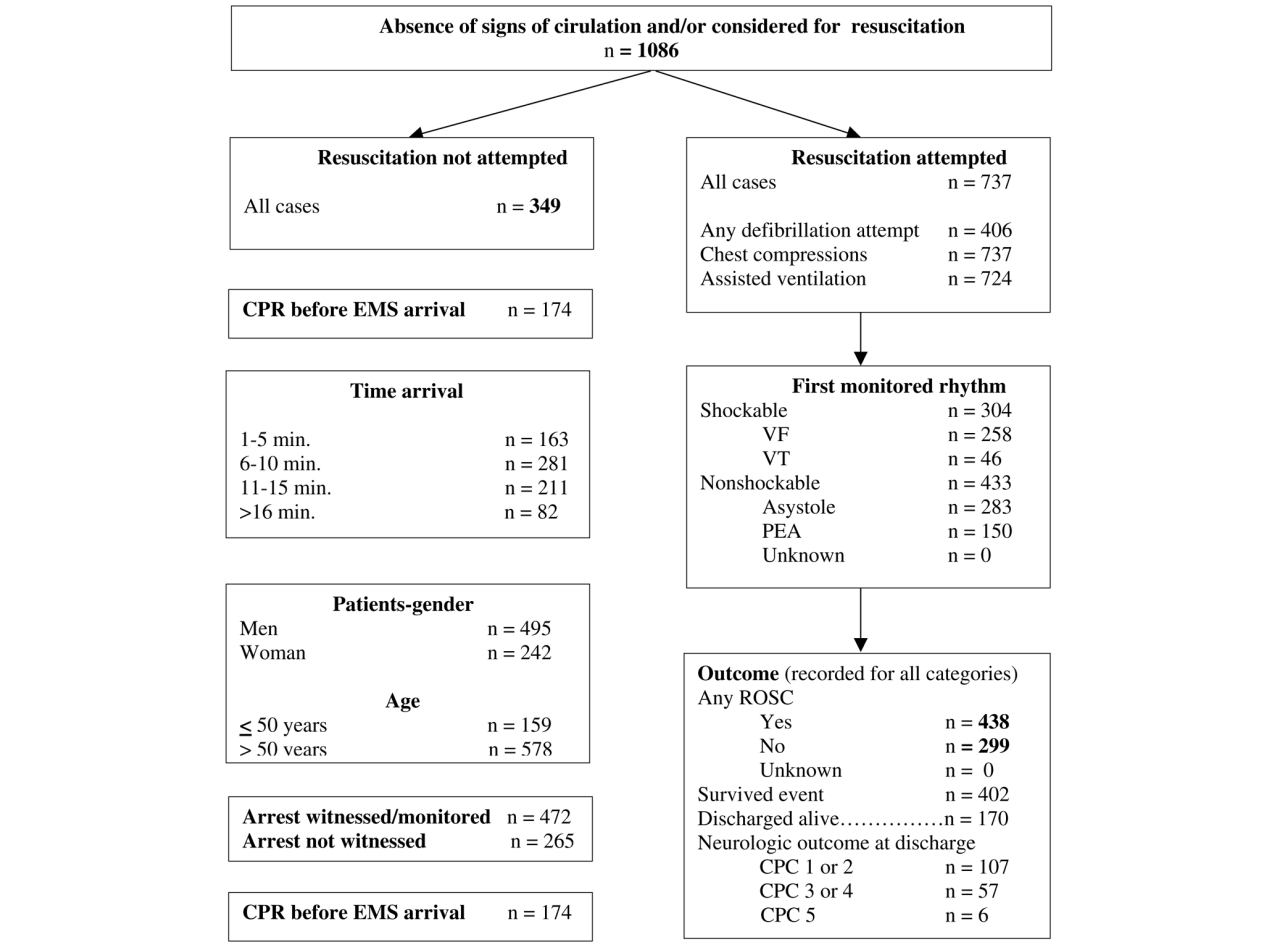
Utstein reporting template for out-of-hospital cardiac arrest obtained over an 8-year period. CPC, cerebral performance category; CPR indicates cardiopulmonary resuscitation; EMS, emergency medicine services; PEA, pulseless electrical activity; ROSC, restoration of spontaneous circulation; VF, ventricular fibrillation; VT, ventricular tachycardia.

During the period of evaluation, our centre was involved in 1,086 emergency interventions in which there was absence of signs of circulation at the start of intervention. Ultimately, 737 patients were resuscitated. ROSC was achieved in 438 patients (59.4%), overall survival to hospital admission occurred in 55% (402 patients) and 170 (23%) patients were discharged alive (Figure 1).

The univariate analysis for ROSC on admission (Table 1) showed that initial PetCO_2_, ventricular fibrillation or pulseless ventricular tachycardia as the initial rhythm, witnessed arrest, bystander-performed CPR, female sex and response time were associated with ROSC. Using the same method, we found that bystander CPR, witnessed arrest, final PetCO_2_, initial PetCO_2 _and resposne time were associated with survival. The initial PetCO_2 _was higher in patients who survived and in those who achieved ROSC (values expressed as kPa [mmHg]; surviving patients: 3.17 [23.8] ± 1.42 [10.7] versus 2.34 [17.6] ± 1.95 [14.7]; ROSC patients 3.13 [23.5] ± 1.65 [12.4] versus 2.54 [19.1] ± 2.43 [18.3]; *P *< 0.001). The final PetCO_2 _(kPa [mmHg]; surviving patients: 3.89 [29.3] ± 1.12 [8.4] versus 1.99 [15.0 mmHg] ± 1.33 [10.0]; ROSC patients: 3.64 [27.4] ± 0.94 [7.1] versus 0.97 [7.3] ± 0.33 [2.5]; *P *< 0.001) was also considerably higher in the surviving and ROSC patients (Table 2).

**Table 1:** Clinical and demographic characteristics for 737 of cardiac arrest patients, according to immediate outcome (ROSC with hospital admission)

	Death in the field (n = 335)	ROSC with hospitalization (n = 402)	*P *value
Male sex (n [%])^a^	242 (72.2%)	253 (62.9%)	0.007
Age (years; mean ± SD)^b^	62.7 ± 15.8	58.8 ± 12.8	0.049
Initial shockable (VF/VT) rhythm (n [%])^a^	92 (27.5%)	212(52.7%)	<0.001
Witnessed arrests (n [%])^a^	176 (52.5%)	294 (73.1%)	0.001
Bystander CPR (n [%])^a^	41 (12.2%)	132 (32.8%)	<0.001
Initial PetCO_2 _(kPa [mmHg]; mean ± SD)^b^	2.6 ± 2.4 (19.6 ± 18.1)	3.1 ± 1.6 (23.5 ± 12.4)	<0.001
Final PetCO_2 _(kPa [mmHg]; mean ± SD)^b^	0.9 ± 0.3 (7.3 ± 2.5)	3.5 ± 0.9 (27.4 ± 7.1)	<0.001
Response time (minutes; mean ± SD)^b^	11.2 ± 4.3	7.8 ± 3.9	<0.001

^a^Fisher test. ^b^Wilcoxon rank sum test. CPR, cardiopulmonary resuscitation; PetCO_2_, partial end-tidal pressure of carbon dioxide; SD, standard deviation; VF, ventricular fibrillation; VT, ventricular tachycardia.

**Table 2:** Clinical and demographic characteristics for the 737 cardiac arrest patients, according to survival (discharge from hospital)

	Death (in the field and in hospital; n = 567)	Survivors (discharge from hospital; n = 170)	*P *value
Male sex (n [%])^a^	384 (67.7%)	111 (65.3%)	0.224
Age (years; mean ± SD)^b^	61.2 ± 14.7	60.2 ± 13.3	0.861
Initial shockable (VF/VT) rhythm (n [%])^a^	209 (36.9%)	95(55.8%)	0.206
Witnessed arrests (n [%])^a^	314 (55.4%)	160 (94.1%)	<0.001
Bystander CPR (n [%])^a^	86 (15.2%)	88 (51.8%)	<0.001
Initial PetCO_2 _(kPa [mmHg]; mean ± SD)^b^	2.4 ± 1.9 (17.6.0 ± 14.7)	3.1 ± 1.4 (23.8 ± 10.7)	<0.001
Final PetCO_2 _(kPa [mmHg]; mean ± SD)^b^	1.9 ± 1.4 (15.0 ± 10.0)	3.9 ± 1.1 (29.3 ± 8.4)	<0.001
Response time (minutes; mean ± SD)^b^	12.7 ± 4.4	6.3 ± 3.2	<0.001

^a^Fisher test. ^b^Wilcoxon rank sum test. CPR, cardiopulmonary resuscitation; PetCO_2_, partial end-tidal pressure of carbin dioxide; SD, standard deviation; VF, ventricular fibrillation; VT, ventricular tachycardia.

The PetCO_2 _value after 20 minutes of advanced life support averaged 0.91 kPa (6.8 mmHg) ± 0.29 kPa (2.2 mmHg) in patients without ROSC and 4.36 kPa (32.8 mmHg) ± 1.11 kPa (8.4 mmHg) in those who achieved ROSC (*P *< 0.001). An end-tidal carbon dioxide value above 1.9 kPa (14.3 mmHg) discriminated between the 402 patients with ROSC and 335 patients without ROSC. When an end-tidal carbon dioxide value of 1.9 kPa (14.3 mmHg) or less was used to predict death, the sensitivity, specificity, PPV and NPV were all 100% (Table 3).

**Table 3:** Performance of various values of PetCO_2 _and duration of CPR for predicting ROSC in all patients with cardiac arrest

PetCO_2_	Cut-off (kPa [mmHg])	n	Min-max (kPa [mmHg])	Mean ± SD (kPa [mmHg])	Sensitivity (%)	Specificity (%)	PPV (%)	NPV (%)	AUROC (95% CI)
Initial	≤1.3 (≤10)	168	0.0–1.3 (0.0–10)	0.68 ± 0.34 (5.1 ± 2.5)	100	50	71	100	0.68 (0.63–0.72)
	>1.3 (>10)	569	1.4–8.7 (10.1–65.4)	3.52 ± 1.93 (26.3 ± 14.5)					
0 to 10 minutes	≤1.6 (≤12.1)	306	0.3–1.6 (2.3–12.1)	0.96 ± 0.34 (7.2 ± 2.6)	100	91	93	100	0.99 (0.99–1.00)
	>1.6 (>12.1)	431	1.7–5.6 (12.2–42.1)	2.79 ± 0.82 (20.9 ± 0.8)					
10 minutes	≤1.3 (≤9.8)	202	0.3–1.3 (2.3–9.8)	0.85 ± 0.31 (6.4 ± 2.1)	100	60	75	100	0.99 (0.98–0.99)
	>1.3 (>9.8)	535	1.4–7.2 (9.9–54.2)	2.89 ± 1.09 (21.7 ± 8.2)					
11–15 minutes	≤1.7 (≤12.8)	333	0.4–1.7 (3.1–12.8)	0.99 ± 0.30 (7.6 ± 2.2)	100	99	99	100	1.00 (0.99–1.00)
	>1.7 (>12.8)	404	1.8–5.5 (12.8–41.4)	3.19 ± 0.77 (23.9 ± 4.8)					
15 minutes	≤1.8 (≤13.6)	328	0.2–1.8 (1.5–13.5)	1.11 ± 0.39 (8.4 ± 2.8)	100	98	98	100	0.99 (0.99–1.00)
	>1.8 (>13.6)	409	1.9–7.7 (13.6–57.9)	3.65 ± 0.98 (27.8 ± 7.4)					
20 minutes	≤1.9 (≤14.3)	335	0.3–1.9 (2.3–14.3)	0.92 ± 0.29 (6.8 ± 2.1)	100	100	100	100	1.00 (1.00–1.00)
	>1.9 (>14.3)	402	2.1–7.8 (14.4–58.7)	4.36 ± 1.11 (33.1 ± 8.4)					
Maximal	≤2.3 (≤17.3)	293	0.7–2.3 (5.3–17.3)	1.58 ± 0.34 (12.1 ± 2.6	100	87	91	100	0.99 (0.99–1.00)
	>2.3 (>17.3)	444	2.4–10.7 (18.1–80.5)	5.12 ± 1.57 (38.4 ± 11.9)					
Final	≤1.7 (≤12.8)	335	0.2–1.7 (1.7–12.8)	0.98 ± 0.33 (7.4 ± 3.2)	100	99	99	100	1.00 (1.00–1.00)
	>1.7 (>12.8)	402	1.9–6.6 (14.3–49.7)	0.98 ± 0.33 (27.8 ± 7.2)					

AUROC, area under the receiver operating characteristic curve; CI, confidence interval; CPR, cardiopulmonary resuscitation; NPV, negative predictive value; PetCO_2_, partial pressure of end-tidal carbon dioxide; PPV, positive predictive value; ROSC, return of spontaneous circulation; SD, standard deviation

A 15-minute PetCO_2 _value of 1.8 kPa (13.5 mmHg) had a sensitivity and NPV of 100%, with high specificity and positive predictive value (98%).

In the patients with nonshockable initial rhythm (pulseless electrical activity), we observed significantly higher initial PetCO_2 _values in comparison with the patients with shockable initial rhythm. On the contrary, in the group of patients who presented with ventricular fibrillation/pulseless tachycardia arrest, there were significantly higher values of PetCO_2 _from the first minute of CPR to the final value (admission to hospital or termination of CPR; Table 4).

**Table 4:** Comparison of characteristics and values of PetCO_2 _between shockable and nonshockable initial rhythm for patients with cardiac arrest

	Shockable (n = 304)	Nonshockable (n = 433)	*P *value
Age (years)	59.5 ± 11.9	60.1 ± 12.9	0.55
Arrival (min [min-max])	8.6 ± 4.5 (1–22)	9.9 ± 4.3 (2–29)	0.03
Initial PetCO_2 _(kPa [mmHg])	2.2 ± 1.3 (16.6 ± 9.8)	3.4 ± 2.4 (25.6 ± 18.1)	<0.001
1 minute PetCO_2 _(kPa [mmHg])	3.3 ± 1.4 (24.8 ± 10.5)	2.8 ± 1.5 (21.1 ± 11.3)	<0.001
Average PetCO_2 _(0 to 10 minutes; kPa [mmHg])	2.3 ± 1.1 (17.3 ± 8.3)	1.8 ± 1.2 (13.5 ± 0.9)	<0.001
10 minute PetCO_2 _(kPa [mmHg])	2.7 ± 1.3 (20.3 ± 10)	2.1 ± 1.3 (15.8 ± 10)	<0.001
11–15 minutes PetCO_2 _(kPa [mmHg])	2.5 ± 1.2 (18.8 ± 9.1)	1.9 ± 1.2 (14.3 ± 9.7)	<0.001
15 minutes PetCO_2 _(kPa [mmHg])	2.9 ± 1.5 (21.8 ± 11.3)	2.2 ± 1.4 (16.5 ± 10.5)	<0.001
20 minutes PetCO_2 _(kPa [mmHg])	3.3 ± 1.8 (24.8 ± 13.5)	2.4 ± 1.9 (18.1 ± 14.3)	<0.001
Max PetCO_2 _(kPa [mmHg])	4.2 ± 2.1 (31.6 ± 15.8)	3.3 ± 2.1 (24.8 ± 15.8)	<0.001
Final PetCO_2 _(kPa [mmHg])	2.9 ± 1.5 (21.8 ± 11.3)	2.1 ± 1.5 (15.8 ± 11.3)	<0.001

'Shockable' was defined as ventricular fibrillation or tachycardia and 'nonshockable' was defined as asystole or pulseless electrical activity. Unless otherwise stated, values are expressed as mean ± standard deviation. PetCO_2_, partial pressure of end-tidal carbon dioxide.

The values of PetCO_2 _in both groups (the group of shockable and the group of nonshockable initial rhythm) were significantly higher in patients with ROSC than in the patients without ROSC (except the PetCO_2 _after 1 minute of CPR in patients with asystole or pulseless electrical activity as initial rhythm). No patients with an initial, average, final, or maximum PetCO_2 _value of less than 1.33 kPa (10 mmHg) was resuscitated (Tables 5 and 6).

**Table 5:** Comparison of characteristics and values of PetCO_2 _between patients with ROSC and without ROSC in shockable initial rhythm in cardiac arrest

	ROSC (n = 211)	Non-ROSC (n = 93)	*P *value
Age (years)	58.6 ± 10.9	61.8 ± 13.6	0.03
Initial PetCO_2 _(kPa [mmHg])	2.7 ± 1.1 (20.3 ± 9.2)	1.8 ± 1.3 (13.5 ± 10)	<0.001
1 minute PetCO_2 _(kPa [mmHg])	3.6 ± 1.3 (27.1 ± 10)	2.6 ± 1.4 (19.6 ± 11)	<0.001
Average PetCO_2 _(0 to 10 minutes; kPa [mmHg])	2.9 ± 0.8 (21.8 ± 6.1)	1.1 ± 0.4 (8.3 ± 3.1)	<0.001
10 minute PetCO_2 _(kPa [mmHg])	3.3 ± 0.9 (24.8 ± 6.8)	1.2 ± 0.5 (9.1 ± 4.2)	<0.001
11 to 15 minute PetCO_2 _(kPa [mmHg])	3.2 ± 0.7 (24.1 ± 5.1)	0.9 ± 0.3 (6.8 ± 2.7)	<0.001
15 minute PetCO_2 _(kPa [mmHg])	3.7 ± 0.9 (27.9 ± 6.8)	1.1 ± 0.4 (8.3 ± 3.4)	<0.001
20 minute PetCO_2 _(kPa [mmHg])	4.3 ± 1.1 (32.3 ± 8.7)	0.9 ± 0.3 (7.1 ± 2.6)	<0.001
Max PetCO_2 _(kPa [mmHg])	5.3 ± 1.5 (39.9 ± 11.3)	1.7 ± 0.6 (12.8 ± 5.5)	<0.001
Final PetCO_2 _(kPa [mmHg])	3.7 ± 0.9 (27.8 ± 6.6)	1.0 ± 0.3 (7.5 ± 2.7)	<0.001

'Shockable' was defined as ventricular fibrillation or pulseless tachycardia. Values are expressed as mean ± standard deviation. PetCO_2_, partial pressure of end-tidal carbon dioxide; ROSC, restoration of spontaneous circulation

**Table 6:** Comparison of characteristics and values of PetCO_2 _between patients with ROSC and without ROSC in nonshockable initial rhythm in cardiac arrest

	ROSC (n = 191)	Non-ROSC (n = 242)	*P *value
Age (years)	59.6 ± 12.9	60.5 ± 12.9	0.45
Initial PetCO_2 _(kPa [mmHg])	3.7 ± 1.9 (27.8 ± 14.3)	3.1 ± 2.6 (23.3 ± 19.6)	0.02
1 minute PetCO_2 _(kPa [mmHg])	2.8 ± 1.6 (21.1 ± 13.2)	2.7 ± 1.4 (20.3 ± 11.2)	0.44
Average PetCO_2 _(0 to 10 minutes; kPa [mmHg])	2.8 ± 0.9 (22.2 ± 6.8)	1.1 ± 0.4 (7.8 ± 3.8)	< 0.001
10 minute PetCO_2 _(kPa [mmHg])	3.3 ± 1.1 (24.8 ± 7.8)	1.2 ± 0.5 (8.2 ± 3.6)	<0.001
Average 11 to 15 minute PetCO_2 _(kPa [mmHg])	3.2 ± 0.8 (24.1 ± 6.3)	1.0 ± 0.3 (7.7 ± 2.6)	<0.001
15 minute PetCO_2 _(kPa [mmHg])	3.6 ± 0.9 (27.1 ± 7.2)	1.1 ± 0.4 (7.9 ± 3.5)	<0.001
20 minute PetCO_2 _(kPa [mmHg])	4.4 ± 1.2 (33.1 ± 9.1)	0.9 ± 0.3 (9.2 ± 2.7)	<0.001
Max PetCO_2 _(kPa [mmHg])	5.4 ± 1.5 (40.1 ± 12.3)	1.8 ± 0.6 (15.6 ± 4.4)	<0.001
Final PetCO_2 _(kPa [mmHg])	3.6 ± 0.9 (27.3 ± 7.1)	0.9 ± 0.3 (7.3 ± 2.5)	<0.001

Nonshockable' was defined as asystole or pulseless electrical activity. Values are expressed as mean ± standard deviation. PetCO_2_, partial pressure of end-tidal carbon dioxide; ROSC, restoration of spontaneous circulation.

After 20 minutes of CPR, PetCO_2 _(regardless of initial rhythm) clearly discriminated between survivors and nonsurvivors in the field (admission to hospital; Tables 7 and 8). In the shockable group PetCO_2 _values above 1.5 kPa (11.3 mmHg; for a positive outcome), and in the nonshockable group values above 1.90 kPa (14.3 mmHg) had a sensitivity, specificity, PPV and NPV values of 100%, and the AUROC was 1.

**Table 7:** Performance of various values of PetCO_2 _and duration of CPR for prediction of ROSC in patients with shockable initial rhythm in cardiac arrest

PetCO_2_	Cut-off (kPa [mmHg])	n	Min-max (kPa [mmHg])	Mean ± SD (kPa [mmHg])	Sensitivity (%)	Specificity (%)	PPV (%)	NPV (%)	AUROC (95% CI)
Initial	≤1.3 (10)	71	0.0–1.3 (0.0–10)	0.69 ± 0.3 (5.2 ± 2.6)	100	76	91	100	0.93 (0.86–0.97)
	>1.3 (10)	233	1.4–8.7 (10.1–65.4)	2.59 ± 1.08 (19.6 ± 8.3)					
0–10 minute (average)	≤1.6 (12.1)	86	0.3–1.6 (2.8–12.1)	0.96 ± 0.33 (7.22 ± 3.1)	100	92	97	100	0.99 (0.99–1.00)
	>1.6 (12.1)	218	1.7–4.8 (12.2–39.1)	2.83 ± 0.77 (21.1 ± 5.8)					
10 minute	≤1.5 (11.3)	72	0.3–1.5 (2.6–11.3)	0.96 ± 0.37 (7.2 ± 2.9)	100	77	91	100	0.99 (0.98–0.99)
	>1.5 (11.3)	232	1.6–5.8 (11.4–43.6)	3.17 ± 0.96 (24.1 ± 7.3)					
11–15 minute (average)	≤1.6 (12.1)	93	0.4–1.6 (3.7–12.1)	0.99 ± 0.28 (7.5 ± 2.1)	100	100	100	100	1.00 (1.00–1.00)
	>1.6 (12.1)	211	1.8–5.5 (13.5–41.4)	3.22 ± 0.74 (24.1 ± 8.3)					
15 minute	≤1.8 (13.5)	92	0.2–1.8 (1.6–13.5)	1.11 ± 0.39 (7.9 ± 2.9)	100	99	100	100	1.00 (1.00–1.00)
	>1.8 (13.5)	212	1.9–7.7 (13.6–59.3)	3.71 ± 0.99 (27.8 ± 7.3)					
20 minute	≤1.5 (11.3)	93	0.3–1.5 (2.8–11.3)	0.95 ± 0.26 (7.3 ± 2.2)	100	100	100	100	1.00 (1.00–1.00)
	>1.5 (11.3)	211	2.1–7.3 (11.4–54.9)	4.33 ± 1.11 (32.3 ± 7.9)					
Max	≤2.3 (17.3)	83	0.7–2.3 (5.26–17.3)	1.56 ± 0.34 (13.2 ± 3.4)	100	89	95	100	0.99 (0.90–1.00)
	>2.3 (17.3)	221	2.4–10.6 (17.4–79.7)	5.23 ± 1.5 (39.2 ± 11.3)					
Final	≤1.5 (11.3)	93	0.3–1.5 (2.6–11.3	1.0 ± 0.32 (7.5 ± 1.9)	100	100	100	100	1.00 (1.00–1.00)
	>1.5 (11.3)	211	1.9–6.3 (11.3–47.4)	3.69 ± 0.94 (27.8 ± 7.1)					

'Shockable' was defined as ventricular fibrillation or tachycardia. AUROC, area under the receiver operating characteristic curve; CI, confidence interval; CPR, cardiopulmonary resuscitation; NPV, negative predictive value; PetCO_2_, partial pressure of end-tidal carbon dioxide; PPV, positive predictive value; ROSC, restoration of spontaneous circulation; SD, standard deviation.

**Table 8:** Performance of various values of PetCO_2 _and duration of CPR for prediction of ROSC in patients with nonshockable initial rhythm in cardiac arrest

PetCO_2_	Cut-off (kPa [mmHg])	n	Min-max (kPa [mmHg])	Mean ± SD (kPa [mmHg])	Sensitivity (%)	Specificity (%)	PPV (%)	NPV (%)	AUROC (95% CI)
Initial	≤1.3 (10)	97	0.0–1.3 (0.0–10)	0.66 ± 0.32 (5.1 ± 2.3)	100	40	57	100	0.61 (0.56–0.67)
	>1.3 (10)	336	1.4–8.4 (10.1–63.2)	4.17 ± 2.2 (30.8 ± 15.8)					
0–10 minute (average)	≤1.6 (12.1)	220	0.4–1.6 (3.4–12.1)	0.97 ± 0.34 (7.3 ± 2.2)	100	91	90	100	0.99 (0.98–0.99)
	>1.6 (12.1)	213	1.7–5.6 (12.2–42.1)	2.74 ± 0.87 (20.6 ± 6.7)					
10 minute	≤1.3 (11)	147	0.3–1.3 (3.6–11)	0.86 ± 0.32 (6.9 ± 2.1)	100	61	67	100	0.98 (0.97–0.99)
	>1.3 (11)	286	1.4–7.2 (11.1–54.2)	2.74 ± 1.14 (21.1 ± 7.9)					
11–15 minute (average)	≤1.7 (12.8)	240	0.4–1.7 (3.7–12.8)	0.99 ± 0.31 (7.3 ± 2.6)	100	99	99	100	1.00 (0.99–1.00)
	>1.7 (12.8)	193	1.9–5.1 (12.9–38.4)	3.15 ± 0.79 (24.1 ± 7.1)					
15 minute	≤1.8 (13.5)	236	0.3–1.8 (2.3–13.5)	1.11 ± 0.39 (7.7 ± 3.3)	100	98	97	100	0.99 (0.99–1.00)
	>1.8 (13.5)	197	1.9–6.1 (13.6–45.9)	3.58 ± 0.97 (27.1 ± 7.4)					
20 minute	≤1.9 (14.3)	242	0.3–1.9 (2.2–14.3)	0.91 ± 0.29 (7.3 ± 2.4)	100	100	100	100	1.00 (1.00–1.00)
	>1.9 (14.3)	191	2.1–7.8 (14.4–58.7)	4.38 ± 1.17 (33.1 ± 7.6)					
Max	≤2.3 (17.3)	210	0.8–2.3 (6.7–17.3)	1.59 ± 0.35 (13.2 ± 3.4)	100	87	86	100	1.00 (1.00–1.00)
	>2.3 (17.3)	223	2.4–10.7 (17.4–80.5)	5.0 ± 1.61 (37.6 ± 15.8)					
Final	≤1.6 (12.3)	239	0.2–1.6 (1.8–12.3)	0.97 ± 0.33 (7.2 ± 2.8)	100	99	98	100	1.00 (1.00–1.00)
	>1.6 (12.3)	194	1.0–6.6 (12.4–49.6)	3.59 ± 0.98 (27.1 ± 7.4)					

'Nonshockable' was defined as asystole or pulseless electrical activity. AUROC, area under the receiver operating characteristic curve; CI, confidence interval; CPR, cardiopulmonary resuscitation; NPV, negative predictive value; PetCO_2_, partial pressure of end-tidal carbon dioxide; PPV, positive predictive value; ROSC, restoration of spontaneous circulation; SD, standard deviation

After 15 minutes of CPR, PetCO_2 _values above 1.8 kPa (13.5 mmHg), in both shockable and nonshockable groups, had sensitivity and NPV of 100%, with acceptable specificity and PPV, and an AUROC of 1 (Tables 7 and 8).

At 20 minutes of CPR, a cut-off point for PetCO_2 _values of 1.5 kPa (13.5 mmHg) yielded sensitivity and NPV of 100% in terms of predicting discharge from hospital in patients with shockable intial rhythm. With a 20-minute PetCO_2 _cut-off of 2.1 kPa (15.8 mmHg), the sensitivity and NPV were 100% in terms of predicting discharge from hospital in patients with nonshockable initial rhythm (Table 9, Table 10).

**Table 9:** Performance of various values of PetCO_2 _and duration of CPR for prediction of survival in patients with shockable initial rhythm in cardiac arrest

PetCO_2_	Cut-off (kPa [mmHg])	n	Min-max (kPa [mmHg])	Mean ± SD (kPa [mmHg])	Sensitivity (%)	Specificity (%)	PPV (%)	NPV (%)	AUROC (95% CI)
Initial	≤1.3 (10)	71	0.0–10 (0.0–1.3)	5.3 ± 1.9 (0.69 ± 0.35)	100	34	40	100	0.73 (0.67–0.78)
	>1.3 (10	233	10.1–65.4 (1.4–8.7)	19.5 ± 6.8 (2.59 ± 1.07)					
0–10 minute (average)	≤1.7 (12.8)	91	0.3–1.7 (2,5–12.8)	0.99 ± 0.36 (7.5 ± 2.1)	100	43	44	100	0.82 (0.78–0.87)
	>1.7 (12.8)	213	1.8–4.8 (12.9–36.1)	2.86 ± 0.76 (21.8 ± 5.2)					
10 minute	≤1.6 (12.1)	82	0.3–1.6 (2.3–12.1)	1.04 ± 0.41 (7.6 ± 3.4)	100	39	42	100	0.82 (0.78–0.87)
	>1.6 (12.1)	222	1.7–5.8 (12.2–43.6)	3.24 ± 0.93 (24.1 ± 7.1)					
11–15 minute (average)	≤1.6 (12.1)	93	0.4–1.6 (3.3–12.2)	0.99 ± 0.28 (7.4 ± 2.3)	100	44	45	100	0.78 (0.73–0.83)
	>1.6 (12.1)	211	1.8–5.5 (12.3–41.4)	3.22 ± 0.74 (24.4 ± 4.9)					
15 minute	≤1.9 (14.3)	94	0.2–1.9 (1.9–14.3)	1.25 ± 0.41 (7.9 ± 2.9)	100	45	45	100	0.78 (0.73–0.83)
	>1.9 (14.3)	210	2.0–7.7 (14.4–57.9)	3.73 ± 0.98 (27.8 ± 7.1)					
20 minute	≤1.5 (11.3)	93	0.3–1.5 (2.8–11.3)	0.95 ± 0.26 (7.2 ± 2.1)	100	44	45	100	0.78 (0.72–0.83)
	>1.5 (11.3)	211	2.1–7.3 (11.4–54.9)	4.33 ± 1.11 (32.3 ± 7.8)					
Max	≤2.5 (18.8)	89	0.7–2.5 (0.7–18.8)	1.62 ± 0.39 (12.4 ± 3.3)	100	42	44	100	0.81 (0.76–0.86)
	>2.5 (18.8)	215	2.6–10.6 (18.9–79.7)	5.3 ± 1.48 (39.9 ± 11.5)					
Final	≤1.5 (11.3)	93	0.3–1.5 (2.7–11.3)	1.0 ± 0.32 (7.5 ± 3.1)	100	44	45	100	0.78 (0.73–0.83)
	>1.5 (11.3)	211	1.9–6.3 (11.4–47.4)	3.69 ± 0.94 (27.8 ± 6.9)					

'Shockable' was defined as ventricular fibrillation or tachycardia. AUROC, area under the receiver operating characteristic curve; CI, confidence interval; CPR, cardiopulmonary resuscitation; NPV, negative predictive value; PetCO_2_, partial pressure of end-tidal carbon dioxide; PPV, positive predictive value; ROSC, restoration of spontaneous circulation; SD, standard deviation.

**Table 10:** Performance of various values of PetCO_2 _and duration of CPR for prediction of survival in patients with nonshockable initial rhythm in cardiac arrest

PetCO_2_	Cut-off (kPa [mmHg])	n	Min-max (kPa [mmHg])	Mean ± SD (kPa [mmHg])	Sensitivity (%)	Specificity (%)	PPV (%)	NPV (%)	AUROC (95% CI)
initial	≤1.3 (10)	97	0.0–1.3 (0.0–10)	0.66 ± 0.32 (5.1 ± 1.9)	100	27	23	100	0.58 (0.52–0.63)
	>1.3 (10)	336	1.4–8.4 (10.1–63.2)	4.17 ± 2.12 (30.9 ± 15.8)					
0–10 min	≤1.7 (12.8)	229	0.4–1.7 (3.5–12.8)	0.99 ± 0.36 (7.1 ± 3.3)	100	64	37	100	0.88 (0.84–0.91)
	>1.7 (12.8)	204	1.8–5.6 (12.9–42.1)	2.79 ± 0.86 (21.5 ± 6.2)					
10 min	≤1.6 (12.1)	199	0.3–1.6 (2.2–12.1)	1.03 ± 0.38 (7.6 ± 3.1)	100	56	32	100	0.87 (0.83–0.91)
	>1.6 (12.1)	234	1.7–7.2 (12.2–54.2)	3.02 ± 1.08 (22.6 ± 6.5)					
11–15 min	≤1.7 (12.8)	239	0.4–1.7 (3.4–12.8)	0.99 ± 0.31 (7.3 ± 2.8)	100	67	39	100	0.86 (0.83–0.90)
	>1.7 (12.8)	194	1.4–5.1 (12.9–38.4)	3.14 ± 0.81 (23.3 ± 6.7)					
15 min	≤1.9 (14.3)	241	0.3–1.9 (2.4–14.3)	1.12 ± 0.41 (7.9 ± 3.8)	100	67	39	100	0.87 (0.84–0.91)
	>1.9 (14.3)	192	2.1–6.1 (14.4–45.9)	3.62 ± 0.94 (28.6 ± 7.5)					
20 min	≤2.1 (15.8)	242	0.3–2.1 (2.3–15.8)	0.91 ± 0.31 (7.2 ± 2.2)	100	68	40	100	0.87 (0.84.0.91)
	>2.1 (15.8)	190	2.3–7.8 (15.9–58.7)	4.39–1.11 (33.1 ± 7.7)					
max	≤2.8 (21.1)	231	0.8–2.8 (6.1–21.1)	1.68 ± 0.44 (13.4 ± 3.5)	100	65	38	100	0.89 (0.86–0.92)
	>2.8 (21.1)	202	2.9–10.7 (21–80.5)	5.26 ± 1.48 (39.1 ± 12.2)					
Final	≤1.6 (12.1)	240	0.2–1.6 (1.9–12.1)	0.97 ± 0.33 (6.9 ± 2.7)	100	67	39	100	0.87 (0.84–0.91)
	>1.6 (12.1)	193	1.9–6.6 (12.2–49.6)	3.06 ± 0.97 (27.1 ± 7.2)					

'Nonshockable' was defined as asystole or pulseless electrical activity. AUROC, area under the receiver operating characteristic curve; CI, confidence interval; CPR, cardiopulmonary resuscitation; NPV, negative predictive value; PetCO_2_, partial pressure of end-tidal carbon dioxide; PPV, positive predictive value; ROSC, restoration of spontaneous circulation; SD, standard deviation.

In multivariate analysis (Table 11), initial, average, 10-minute, 15-minute, 20-minute, maximum and final values of PetCO_2_, shockable initial rhythm (ventricular fibrillation or tachycardia), witnessed arrest, bystander-performed CPR, female sex and arrival time were associated with improved ROSC. Using the same method we found that bystander CPR, witnessed arrest, shockable initial rhythm, initial, average, 10-minute, 15-minute, 20-minute, maximum and final PetCO_2 _values, and arrival time were associated with improved survival (Table 12).

**Table 11:** Variables associated with ROSC in cardiac arrest

Variable	OR (95% CI)	*P *value
Intial rhythm (VF/VT)	2.13 (1.17–4.22)	0.02
Female sex	1.58 (1.14–1.87)	0.04
Time arrival	1.69 (1.37–2.56)	0.01
Witness	1.65 (1.29–3.14)	0.02
Bystander CPR	3.26 (1.89–8.51)	0.01
Initial PetCO_2_	21.68 (9.72–38.37)	<0.0001
Average PetCO_2_	19.48 (7.53–33.86)	<0.001
10 minute PetCO_2_	14.37 (6.65–28.63)	<0.001
15 minute PetCO_2_	17.41 (7.62–24.57)	<0.001
20 minute PetCO_2_	24.86 (10.11–42.73)	<0.001
Max PetCO_2_	12.23 (4.83–23.64)	<0.001
Final PetCO_2_	18.07 (6.93–28.34)	<0.001

CI, confidence interval; CPR, cardiopulmonary resuscitation; OR, odds ratio; PetCO_2_, partial pressure of end-tidal carbon dioxide; ROSC, restoration of spontaneous circulation; VF, ventricular fibrillation; VT, ventricular tachycardia.

**Table 12:** Variables associated with survival in cardiac arrest

Variables	OR (95% CI)	*P *value
Initial rhythm (VF/VT)	1.86 (1.26–3.11)	<0.001
Arrival time	1.39 (1.33–1.60)	0.01
Witness	9.98 (2.89–34.44)	<0.0001
Bystander CPR	5.05 (2.24–11.39)	<0.0001
Intial PetCO_2_	1.93 (1.48–3.75)	0.018
Average PetCO_2_	2.31 (1.45–4.86)	<0.001
10 min PetCO_2_	2.11 (1.27–4.16)	0.001
15 min PetCO_2_	2.47 (1.33–5.21)	<0.001
20 min PetCO_2_	3.85 (1.71–8.34)	<0.001
Final PetCO_2_	2.37 (1.67–3.37)	<0.001

CI, confidence interval; CPR, cardiopulmonary resuscitation; OR, odds ratio; PetCO_2_, partial pressure of end-tidal carbon dioxide; VF, ventricular fibrillation; VT, ventricular tachycardia.

## Discussion

Presenting the European perspective, Scogvoll and coworkers [14] reported that the annual incidence of attempted CPR ranged from 33 to 71 per 100,000 inhabitants. Sudden cardiac death accounts for approximately 1000 lives per day in the USA [5]. In the majority of cases, CPR and other treatment efforts are unsuccessful, and the patient was eventually pronounced dead. A number of clinical indicators can be used to determine when those efforts should be terminated [15-18]. Morrison and colleagues [12] described a clinical decision rule for termination of resuscitation (TOR), which was designed to help emergency medical services to determine whether to terminate resuscitative efforts in the setting of out-of-hospital cardiac arrest. In that Canadian study, the investigators sought to validate their previously proposed prediction rule, namely that TOR should be considered if spontaneous circulation does not return before transport is initiated, if no automatic external defibrillator (AED) shocks are given before transport is initiated, and if arrest was not witnessed by emergency personnel. This simple prediction rule has 99.5% PPV and a specificity of 90.2%, and may be useful for providing supplementary guidance in the field [17]. However, a rule cannot determine, for example, how long to continue resuscitation efforts before declaring 'no ROSC'. Decisions about TOR continue to cause difficulties for health care professionals. Current guidelines provide some information on underlying principles, but they do not include a objective, clear and numerical decision rule regarding TOR.

Several animal and clinical studies suggest that the PetCO_2 _can be used to determine when resuscitation should be ceased. Investigators have suggested that there is a close correlation between PetCO_2 _and cardiac output, stroke volume, and coronary and cerebral perfusion pressure during CPR. Kalenda [19] first reported a decrease in PetCO_2 _in patients who could not be resuscitated, and a significant rise in PetCO_2 _in those patients in whom ROSC could be achieved.

Falk and coworkers [20] found that PetCO_2 _decreased from mean of 1.4% before arrest to 0.4% after the onset of cardiac arrest. It then increased with CPR and ROSC. Sanders and colleagues [21] found that the end-tidal carbon dioxide level predicted successful resuscitation after in hospital and out-of-hospital cardiac arrest. The average, initial, final, maximum and minimum values of PetCO_2 _were all higher in resuscitated patients. No patient with an average PetCO_2 _value of less than 1.33 kPa (10 mmHg) was resuscitated.

Callaham and Barton [22] found that the four patients who had initial and later PetCO_2 _values of less than 1.33 kPa (10 mmHg) were all resuscitated. These data and similar reports of ROSC after prolonged resuscitative attempts [23] with low PetCO_2 _values may account for the reluctance of the scientific community to incorporate PetCO_2 _in Utstein-style reports and resuscitation algorithms. In a landmark prospective study, Levine and colleagues [8] observed 150 patients suffering cardiac arrest and measured PetCO_2 _using a mainstream capnometer. They compared 20-minute PetCO_2 _and initial values and concluded that initial values are unreliable in predicting mortality. The 20-minute values of PetCO_2 _were promising and more reliable in predicting mortality. Values less then 1.33 kPa (10 mmHg) after 20 minutes of CPR were incompatible with survival, and the authors are of the opinion that this could be helpful in deciding when to stop resuscitation efforts. We established the relationship between PetCO_2 _and prognosis in prehospital CPR in our previous studies [5,24]. In the second study [24], we confirmed that PetCO_2 _and mean arterial pressure values are prognostic for the outcome of out-of-hospital cardiac arrest. During a cardiac arrest, PetCO_2 _can be considered an indirect parameter for the evaluation of cardiac output in the prehospital setting, together with mean arterial pressure, when spontaneous circulation is restored.

Our study is the largest prospective study of the predictive value of PetCO_2 _measurement for ROSC and survival, and includes 737 victims of out-of-hospital sudden cardiac arrest. We confirmed that bystander CPR, witnessed arrest, shockable initial rhythm, initial, average, 10-minute, 15-minute, 20-minute, maximum and final values of PetCO_2 _and arrival time were all associated with improved ROSC and survival.

We found that PetCO_2 _values above 1.9 kPa (14.3 mmHg) measured after 20 minutes of resuscitation identified patients with ROSC with 100% sensitivity, specificity, PPV and NPV. No patients with initial, average, final and maximum PetCO_2 _values of less than 1.33 kPa (10 mmHg) was resuscitated. With a cut-off point of 20-minute PetCO_2 _value at 1.5 kPa (13.5 mmHg) in patients with shockable initial rhythm and a cut-off point at 2.1 kPa (15.8 mmHg) in patients with nonshockable initial rhythm, sensitivity and NPV were 100% in predicting discharge from hospital.

In nonshockable rhythm we found higher initial values and lower values after 1 minute of CPR. In our previous study [25] we confirmed PetCO_2 _to be markedly elevated during the first minute of CPR in asphyxial cardiac arrest. This study therefore confirmed the findings of studies that used animal models in which cardiopulmonary arrest was induced by asphyxia. In this study the PetCO_2 _values during CPR were initially high, then decreasing to subnormal levels and then increasing again to near-normal levels in patients with ROSC. This pattern of PetCO_2 _change is different from the pattern observed in cardiac arrest caused by venticular fibrillation, because cardiac arrest from venticular fibrillation results in an abrupt cessation of cardiac output and pulmonary blood flow. We concluded that, during the period of asphyxia, continued cardiac output before cardiac arrest permits continued delivery of carbon dioxide to the lungs, which (in the absence of exhalation) results in higher alveolar carbon dioxide levels. This is reflected in increased PetCO_2 _when ventilation is resumed.

Our findings in patients with shockable initial rhythm confirmed the view of Levine and coworkers [8] that the data from their study (PetCO_2 _in patients with pulseless electrical activity) can be extended to all types of cardiac arrest. Sehra and coworkers [26], in a human model of ventricular fibrillation, confirmed that PetCO_2 _can predict severity of ventricular fibrillation cardiac arrest and efficacy of CPR in this type of cardiac arrest. Our findings in shockable group possible indirectly confirm the three-phase, time-dependent concept of cardiac arrest due to ventricular fibrillation [26]. PetCO_2 _values under 1.5 kPa (11.3 mmHg) after 20 minutes of CPR (or less that 1.8 kPa [13.5 mmHg] after 15 minutes of CPR) are incompatible with ROSC. This is time of the end of haemodynamic phase of CPR. Possibly, these values represent irreversible hemodynamic collapse, with inadequate coronary or myocardial perfusion pressure, or they may represent perfusion pressures supplied too late (after the haemodynamic phase), with consequent irreversible tissue damage [27,28].

Our prehospital data, combined with the findings of other investigators, provide strong support for a resuscitation thresholds of PetCO_2 _1.33 kPa (10 mmHg) initially and 1.9 kPa (14.3 mmHg) after 20 minutes of CPR in the field. The initial values of PetCO_2 _are not influenced by medications used during CPR, and values at 20 minutes reflect the patient's 'response' to resuscitation efforts. We recommend initial and 20-minute (final PetCO_2_) to be ranked in Utstein-style reports. The objectives of this approach are to assess the initial condition of the patient in the setting of nontraumatic normothermic cardiac arrest, and to optimize the reliability of PetCO_2 _in predicting survival in such patients.

Our finding are potentially important, especially in emergency medical system that do not include physicians. The results of the study confirm that PetCO_2 _can play a pivotal role in the multifactorial decision-making process of whether to discontinue resuscitative efforts. Application of our findings could improve clinical prediction rules for TOR in the field and reduce the number of patients with cardiac arrest who undergo prolonged, futile resuscitation efforts; furthermore, they may reduce transportation of patients with refractory cardiac arrest to the hospital. For the health care system, there is less cost involved in TOR in the field than in the transfer of the patient to the hospital [12,29,30].

## Conclusion

Measurements of PetCO_2 _should be used to predict nonsurvival of patients with cardiopulmonary arrest. End-tidal carbon dioxide levels should be monitored during CPR and should be regarded as having prognostic value for determining the outcome of resuscitative efforts. The results can inform decisions regarding when advanced cardiac life support can be discontinued, thus decreasing costs and dilemmas to resuscitation teams. Based on our findings, we believe that end-tidal carbon dioxide monitoring should be incorporated into advanced cardiac life support algorithms and ranked in Utstein-style reports to provide insight into the condition of patients suffering cardiac arrest.

## Key messages

• A PetCO_2 _level of 1.9 kPa (14.3 mmHg) or less measured 20 minutes after the initiation of advanced cardiac life support accurately predicts death in patients with nonshockable initial rhythm who are suffering cardiac arrest.

• When a 20-minute PetCO_2 _value of 1.5 kPa (11.3 mmHg) or less was used as a screening test to predict death in patients with shockable rhythm, the sensitivity, specificity, PPV and NPV were all 100%.

• Values of PetCO_2 _less than 1.5 kPa (11.3 mmHg) after 20 minutes of CPR (or <1.8 kPa [<13.5 mmHg] after 15 minutes of CPR) are incompatible with ROSC.

• End-tidal carbon dioxide levels should be monitored during CPR, and should be regarded as having prognostic value in predicting the outcome of resuscitative efforts and informing decisions regarding TOR.

## Abbreviations

AUROC: area under the ROC curve; CPC: cerebral performance category; CPR: cardiopulmonary resuscitation; ICU: intensive care unit; NPV: negative predictive value; PetCO_2_: partial pressure of end-tidal carbon dioxide; PPV: positive predictive value; ROC: receiver operating characteristic; ROSC: return of spontaneous circulation; TOR: termination of resuscitation.

## Competing interests

The authors declare that they have no competing interests.

## Authors' contributions

MK participated in designing the study, collection and analysis of data, and helped to draft the manuscript. MK participated in designing the study, and collection and statistical analysis of data. PK participated in designing the study and helped to draft the manuscript. ŠG participated in designing the study, collection and analysis of data, revised the manuscript for important intellectual content and helped to draft the manuscript. All authors read and approved the final version of the manuscript.
